# Tópicos Emergentes em Insuficiência Cardíaca: Abordagem Contemporânea da Insuficiência Cardíaca Avançada

**DOI:** 10.36660/abc.20201194

**Published:** 2020-12-01

**Authors:** Fabiana G. Marcondes-Braga, Jefferson L. Vieira, João David de Souza, Gustavo Calado, Silvia Moreira Ayub-Ferreira, Fernando Bacal, Nadine Clausell

**Affiliations:** 1 Hospital das Clínicas Faculdade de Medicina Universidade de São Paulo São PauloSP Brasil Instituto do Coração do Hospital das Clínicas da Faculdade de Medicina da Universidade de São Paulo (InCor/HCFMUSP),São Paulo, SP - Brasil; 2 Hospital do Coração de Messejana FortalezaCE Brasil Hospital do Coração de Messejana, Fortaleza, CE - Brasil; 3 Pontifícia Universidade Católica de Campinas CampinasSP Brasil Pontifícia Universidade Católica de Campinas (PUCC), Campinas, SP - Brasil; 4 Hospital de Clínicas de Porto Alegre Porto AlegreRS Brasil Hospital de Clínicas de Porto Alegre, Porto Alegre, RS - Brasil

**Keywords:** Insuficiência Cardíaca Avançada, Terapias Avançadas, Transplante Cardíaco, Suporte Circulatório Mecânico, Cuidados Paliativos

## Definição

O termo insuficiência cardíaca (IC) avançada define um perfil de pacientes com sintomas graves, descompensações recorrentes e disfunção cardíaca progressiva a despeito da máxima terapêutica instituída.^1^ Pacientes que permanecem gravemente sintomáticos ou em classe funcional (CF) IV persistente podem ser candidatos a terapias avançadas, como transplante cardíaco (TC), assistência circulatória mecânica (ACM) ou cuidados paliativos. Vale ressaltar que algumas comorbidades, como as doenças pulmonar, renal e hepática, também são consideradas determinantes de pior prognóstico na IC crônica grave; na presença de alguma delas, os pacientes devem ser avaliados para possível indicação de terapias avançadas da IC.

### Avaliação prognóstica

**Figura 1:**
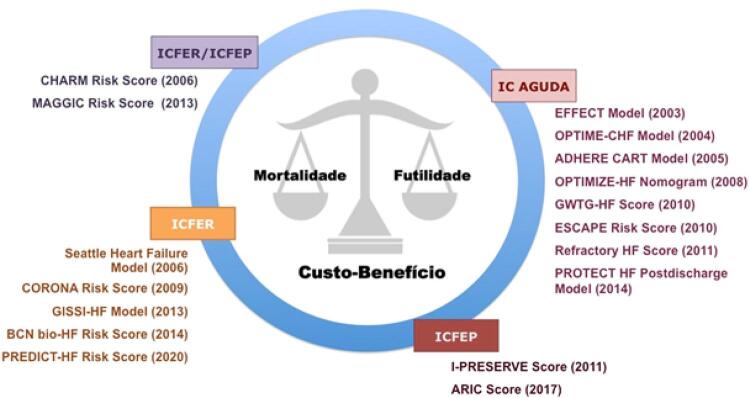
– Escores de risco para insuficiência cardíaca. ADHERE CART: Acute Decompensated Heart Failure National Registry Classification and Regression Tree Analysis; ARIC: Atherosclerosis Risk in Communities; BCN bio-HF: Barcelona Bio-Heart Failure; CHARM: Candesartan in Heart Failure Assessment of Reduction in Mortality and Morbidity; CORONA: Controlled Rosuvastatin Multinational; EFFECT: Enhanced Feedback for Effective Cardiac Treatment; ESCAPE: Evaluation Study of Congestive Heart Failure and Pulmonary Artery Catheterization Effectiveness; GISSI-HF: Gruppo Italiano per lo Studio della Streptochinasi nell’Infarto Miocardico-Heart Failure; GWTG-HF: Get With the Guidelines–Heart Failure; I-PRESERVE: Predicting death for severe acute respiratory distress syndrome on venovenous extracorporeal membrane oxygenation; IC: insuficiência cardíaca; ICFEP: insuficiência cardíaca com fração de ejeção preservada; ICFER: insuficiência cardíaca com fração de ejeção reduzida; MAGGIC: Meta-Analysis Global Group in Chronic Heart Failure; OPTIME-CHF: Outcomes of a Prospective Trial of Intravenous Milrinone for Exacerbations of Chronic Heart Failure; OPTIMIZE-HF: Organized Program to Initiate Lifesaving Treatment in Hospitalized Patients With Heart Failure; PREDICT-HF: Prognostic Models Derived in PARADIGM-HF and Validated in ATMOSPHERE and the Swedish Heart Failure Registry to Predict Mortality and Morbidity in Chronic Heart Failure; PROTECT HF: Placebo-Controlled Randomized Study of the Selective A1 Adenosine Receptor Antagonist Rolofylline for Patients Hospitalized With Acute Decompensated Heart Failure and Volume Overload to Assess Treatment Effect on Congestion and Renal Function.

Existem diversos escores de risco na IC ( Figura 1 ); cada escore foi desenvolvido a partir de coortes específicas, incluindo aquelas com IC aguda, IC com fração de ejeção reduzida e/ou IC com fração de ejeção preservada. Dentre os escores CHARM ( *Candesartan in Heart Failure Assessment of Reduction in Mortality and Morbidity* ), GISSI-HF ( *Gruppo Italiano per lo Studio della Streptochinasi nell’Infarto Miocardico-Heart Failure* ), MAGGIC ( *Meta-Analysis Global Group in Chronic Heart Failure* ) e SHFM ( *Seattle Heart Failure Model* ), o MAGGIC parece apresentar o melhor poder discriminatório para mortalidade em um ano.^2^ Outros escores de risco propostos para ACM de curta e longa permanência, como o SAVE ( *Survival After Veno-Arterial Extracorporeal Membrane Oxygenation* ) e o Escore de Risco HeartMate II, respectivamente, são úteis na seleção de pacientes, embora restritos a dispositivos específicos. Recentemente, o escore PREDICT-HF ( *Prognostic Models Derived in PARADIGM-HF and Validated in ATMOSPHERE and the Swedish Heart Failure Registry to Predict Mortality and Morbidity in Chronic Heart Failure* ) foi criado com dados do estudo PARADIGM-HF ( *Angiotensin–Neprilysin Inhibition versus Enalapril in Heart Failure* ) buscando retratar o manejo contemporâneo da IC e aguarda validação prospectiva.^3^

### Manejo da IC avançada no cenário agudo

#### Manejo da congestão

O manejo da congestão continua sendo um desafio e exige a associação de diferentes estratégias: diurético endovenoso em doses elevadas; associação de diferentes classes de diuréticos; solução salina hipertônica; ultrafiltração e diálise peritoneal.^4^

Embora poucas inovações tenham surgido nesse campo, evidências recentes apontam um impacto da monitorização da congestão no prognóstico de pacientes com IC. Estudos de monitorização não-invasiva por telemonitoramento mostraram benefício sobre tempo de hospitalização e morte por todas as causas;^5^ resultados similares foram observados com o dispositivo implantável CardioMEMS™ HF System, que fornece monitorização direta da artéria pulmonar. O CardioMEMS™ mostrou-se eficaz e seguro em estudos de “vida real”, de custo-efetividade e de pós-comercialização,^6^ com achados semelhantes em centros europeus;^7^ trata-se de uma estratégia promissora com potencial a ser acrescentada à prática clínica.

#### Manejo do choque cardiogênico

**Figura 2:**
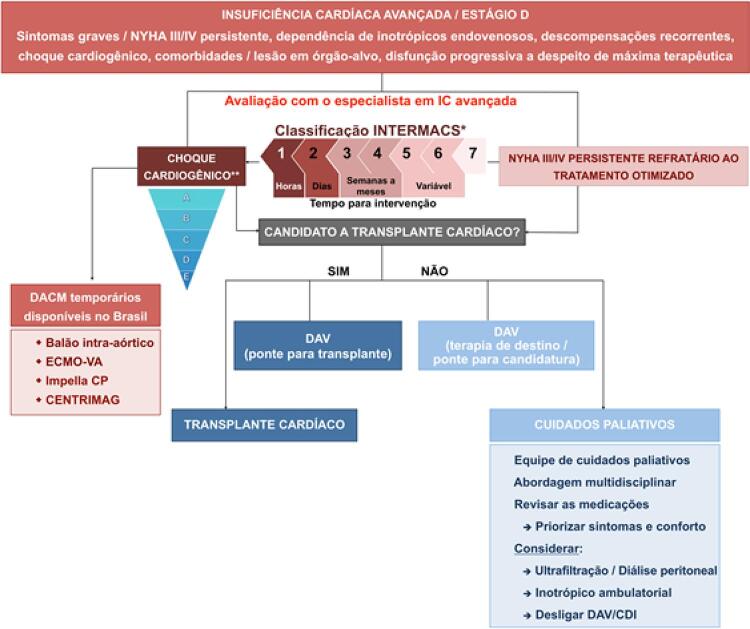
– Algoritmo de tratamento do paciente com insuficiência cardíaca avançada. *Classificação clínica de pacientes com insuficiência cardíaca avançada da Interagency Registry for Mechanically Assisted Circulatory Support (INTERMACS). Perfil 1: choque cardiogênico grave; Perfil 2: declínio progressivo, apesar do uso de inotrópico; Perfil 3: estável às custas de inotrópico endovenoso; Perfil 4: internações frequentes; Perfil 5: em casa, intolerante aos esforços; Perfil 6: limitação aos esforços; Perfil 7: NYHA III. **Classificação de choque cardiogênico proposta pela Society for Cardiovascular Angiography and Interventions (SCAI). Estágio A: “sob risco “ de choque; Estágio B: “início” do choque; Estágio C: choque “clássico”; Estágio D: choque em “deterioração”; Estágio E: “extremo”. Adaptado de Baran DA, et al. SCAI clinical expert consensus statement on the classification of cardiogenic shock. Catheter Cardiovasc Interv. 2019; 94: 29– 37. doi:10.1002/ccd.28329. *CDI: cardiodesfibrilador implantável; CF: classe funcional; DACM: dispositivos de assistência circulatória mecânica; DAV: dispositivo de assistência ventricular implantável; ECMO-VA: circuito de oxigenação por membrana extracorpórea venoarterial; IC: insuficiência cardíaca; NYHA: New York Heart Association.*

Recentemente, a Sociedade de Angiografia Cardiovascular e Intervenção (SCAI) propôs uma classificação do choque cardiogênico (CC) visando estabelecer uma linguagem comum para facilitar a identificação das diferentes fases do choque e planejar o manejo apropriado. As cinco fases dessa classificação permitem uma definição hemodinâmica simples, oportunizando maior discriminação dos estágios da classificação INTERMACS ( *Interagency Registry for Mechanically Assisted Circulatory Support* ).^8^ ( Figura 2 )

Nos últimos anos, estratégias focadas em atuação precoce no curso do CC, envolvendo avaliação multidisciplinar ( *shock team* ), têm destacado o papel do especialista em IC avançada coordenando as decisões terapêuticas com agilidade.^9^ As drogas vasoativas (DVAs) devem ser usadas de maneira a suportar minimamente o perfil hemodinâmico/metabólico, com a recomendação ainda vigente de uso de terapia combinada em baixa dose, com vistas a reduzir dano tecidual. Uma revisão sistemática recente sugere não haver superioridade entre as DVAs, mas sinaliza a importância da estratégia precoce focada em objetivo específico ( *early goal-directed strategy* ) buscando estabilização hemodinâmica dentro de tempos predeterminados.^10^ O escalonamento de drogas deve servir de alerta para a mudança de estratégia em direção ao uso de ACM para evitar a espiral de dano hemodinâmico/metabólico.

Os dispositivos de curta permanência fornecem suporte uni ou biventricular para situações clínicas, como CC, IC aguda, intervenção coronária de alto risco ou parada cardíaca.^11^ Os dispositivos mais utilizados são o balão intra-aórtico (BIA), o Impella^®^, o TandemHeart^®^ e o circuito de oxigenação por membrana extracorpórea venoarterial (ECMO-VA).^4^ Embora esses dispositivos previnam a espiral de dano hemodinâmico/metabólico com sucesso, não há evidência de benefício sobre a mortalidade no CC.^12^ Além disso, estudos observacionais recentes sugeriram maiores taxas de eventos adversos e custos com o Impella^®^ do que com o BIA.^13^ Apesar das limitações, o BIA ainda é o dispositivo de curta permanência mais utilizado no CC.

No campo da pesquisa clínica, o sistema de assistência ventricular intravascular NuPulseCV (iVAS) é um dispositivo minimamente invasivo que fornece contrapulsação ambulatorial de longa permanência por meio de um balão implantável inserido pela artéria subclávia e controlado por uma unidade externa.^14^ O iVAS supera muitas limitações do BIA, sendo uma opção promissora na IC avançada.

## Terapias avançadas

As características dos candidatos a terapias avançadas para IC, como TC e dispositivos de assistência ventricular implantáveis (DAV), mudaram muito ao longo dos anos, tornando o processo de seleção mais complexo. A seguir, abordaremos progressos e desafios no campo das terapias avançadas para IC.

Com relação ao TC, que é o tratamento de escolha para IC avançada,^15^ estratégias para aumentar o número de doadores efetivos tem sido aventadas; de fato, nos Estados Unidos, a UNOS recentemente mudou sua política de alocação de órgãos.^16^ Considerando que a sobrevida pós-transplante é pior com ECMO-VA pré-operatória do que com DAV, o novo sistema atribui maior prioridade aos pacientes com ACM de curta duração, enquanto aqueles com DAV ou inotrópicos são priorizados em um *status* inferior. No Brasil, mudanças semelhantes também estão sendo adotadas em alguns estados. Outra proposta recente é de substituir o peso corporal por massa cardíaca prevista ( *predicted heart mass* , PHM) como ferramenta ideal na avaliação da desproporção de tamanho entre doador-receptor. Estudos têm demonstrado a superioridade da PHM na predição de disfunção primária do enxerto e mortalidade pós-transplante comparada com peso, altura ou índice de massa corporal,^17^ além de predizer recuperação do acoplamento ventrículo-arterial pulmonar após TC.^18^ Por fim, o advento de antivirais de ação direta no tratamento da hepatite C crônica, como o Sofosbuvir, agora permite a alocação de órgãos de doadores infectados em receptores não infectados.^19^

No campo dos DAVs, a incorporação do HeartMate 3™ resultou em benefício clínico expressivo, com redução significativa nas taxas de arritmia ventricular, reinternações e eventos adversos de hemocompatibilidade (sangramento, trombose e acidente vascular cerebral).^20^ Futuros avanços devem envolver a miniaturização dos DAVs e a criação de um sistema totalmente intracorpóreo.

Finalmente, os cuidados paliativos têm se mostrado uma estratégia indispensável no manejo da IC avançada, assumindo protagonismo maior nos casos inelegíveis para TC ou DAVs. O uso intermitente de ultrafiltração, diálise peritoneal ou inotrópicos são estratégias possíveis em regime hospitalar, hospital-dia ou mesmo domiciliar visando ao alívio de sintomas.^1^

## Lista de Participantes do Heart Failure Summit Brazil 2020 / Departamento de Insuficiência Cardíaca - DEIC/SBC

Aguinaldo Freitas Junior, Andréia Biolo, Antonio Carlos Pereira Barretto, Antônio Lagoeiro Jorge, Bruno Biselli, Carlos Eduardo Montenegro, Denilson Campos de Albuquerque, Dirceu Rodrigues de Almeida, Edimar Alcides Bocchi, Edval Gomes dos Santos Júnior, Estêvão Lanna Figueiredo, Evandro Tinoco Mesquita, Fabiana G. Marcondes-Braga, Fábio Fernandes, Fabio Serra Silveira, Felix José Alvarez Ramires, Fernando Atik, Fernando Bacal, Flávio de Souza Brito, Germano Emilio Conceição Souza, Gustavo Calado de Aguiar Ribeiro, Humberto Villacorta Jr., Jefferson Luis Vieira, João David de Souza Neto, João Manoel Rossi Neto, José Albuquerque de Figueiredo Neto, Lídia Ana Zytynski Moura, Livia Adams Goldraich, Luís Beck-da- Silva, Luís Eduardo Paim Rohde, Luiz Claudio Danzmann, Manoel Fernandes Canesin, Marcelo Bittencourt, Marcelo Westerlund Montera, Marcely Gimenes Bonatto, Marcus Vinicius Simões, Maria da Consolação Vieira Moreira, Miguel Morita Fernandes da Silva, Monica Samuel Avila, Mucio Tavares de Oliveira Junior, Nadine Clausell, Odilson Marcos Silvestre, Otavio Rizzi Coelho Filho, Pedro Vellosa Schwartzmann, Reinaldo Bulgarelli Bestetti, Ricardo Mourilhe Rocha, Sabrina Bernadez Pereira, Salvador Rassi, Sandrigo Mangini, Silvia Marinho Martins, Silvia Moreira Ayub Ferreira, Victor Sarli Issa.
