# Supplementary material for: Tópicos Emergentes em Insuficiência Cardíaca: Abordagem Contemporânea da Insuficiência Cardíaca Avançada
Source: Arq Bras Cardiol. 2020 Dec 1;115(6):1193–6. [Article in Portuguese] doi: 10.36660/abc.20201194 (PMC8133710; doi:10.36660/abc.20201194)
Supplement: Translation

# Translation: Emerging Topics in Heart Failure: Contemporaneous Management of Advanced Heart Failure

**Authors:** Fabiana G. Marcondes-Braga, Jefferson L. Vieira, João David de Souza, Gustavo Calado, Silvia Moreira Ayub-Ferreira, Fernando Bacal, Nadine Clausell

**Affiliations:** 1 Brazil Instituto do Coração do Hospital das Clínicas da Faculdade de Medicina da Universidade de São Paulo (InCor/HCFMUSP), São Paulo, SP - Brazil; 2 Brazil Hospital do Coração de Messejana, Fortaleza, CE - Brazil; 3 Brazil Pontifícia Universidade Católica de Campinas (PUCC), Campinas, SP - Brazil; 4 Brazil Hospital de Clínicas de Porto Alegre, Porto Alegre, RS - Brazil

**Keywords:** Advanced Heart Failure, Advanced Therapies, Heart Transplant, Mechanical Circulatory Support, Paliative Care

## Definition

Advanced heart failure (HF) is a condition characterized by persistent severe HF symptoms, frequent episodes of decompensation, and progressive cardiac dysfunction despite optimal evidence-based treatment.^1^These patients may be candidates for advanced therapies, such as heart transplantation (HT), mechanical circulatory support (MCS), and/or palliative care. It should be pointed out that some comorbidities, including pulmonary disease and liver and kidney dysfunction, are now included as possible major determinants of poor prognosis and should be considered during patient evaluation for advanced HF therapies.

### Prognosis and risk scores

There are several risk scores for predicting outcomes in HF populations ( Figure 1 ); each model has been developed for use in specific cohorts, including those with acute HF, HF with reduced ejection fraction, and/or HF with preserved ejection fraction. The MAGGIC (Meta-Analysis Global Group in Chronic Heart Failure) score seems to have better accuracy than the CHARM (Candesartan in Heart Failure Assessment of Reduction in Mortality and Morbidity), GISSI-HF (Gruppo Italiano per lo Studio della Streptochinasi nell’Infarto Miocardico-Heart Failure), and SHFM (Seattle Heart Failure Model) scores for predicting 1-year mortality.^2^Other risk stratification tools for short- and long-term MCS, such as the SAVE (Survival After Veno-Arterial Extracorporeal Membrane Oxygenation) and HeartMate II risk scores, respectively, may be helpful in patient selection, but are restricted to specific devices. Recently, the PREDICT-HF (Prognostic Models Derived in PARADIGM-HF and Validated in ATMOSPHERE and the Swedish Heart Failure Registry to Predict Mortality and Morbidity in Chronic Heart Failure) score used data from the PARADIGM-HF (Angiotensin–Neprilysin Inhibition versus Enalapril in Heart Failure) trial to develop a prognostic model for patients receiving contemporary evidence-based therapies for HF. It has yet to be validated.^3^

**Figure 1:** – Risk scores for heart failure. ADHERE CART: Acute Decompensated Heart Failure National Registry Classification and Regression Tree Analysis; ARIC: Atherosclerosis Risk in Communities; BCN bio-HF: Barcelona Bio-Heart Failure; CHARM: Candesartan in Heart Failure Assessment of Reduction in Mortality and Morbidity; CORONA: Controlled Rosuvastatin Multinational; EFFECT: Enhanced Feedback for Effective Cardiac Treatment; ESCAPE: Evaluation Study of Congestive Heart Failure and Pulmonary Artery Catheterization Effectiveness; GISSI-HF: Gruppo Italiano per lo Studio della Streptochinasi nell’Infarto Miocardico-Heart Failure; GWTG-HF: Get With the Guidelines–Heart Failure; HF: heart failure; HFPEF: heart failure with preserved ejection fraction; HFREF: heart failure with reduced ejection fraction; I-PRESERVE: Predicting death for severe acute respiratory distress syndrome on venovenous extracorporeal membrane oxygenation; MAGGIC: Meta-Analysis Global Group in Chronic Heart Failure; OPTIME-CHF: Outcomes of a Prospective Trial of Intravenous Milrinone for Exacerbations of Chronic Heart Failure; OPTIMIZE-HF: Organized Program to Initiate Lifesaving Treatment in Hospitalized Patients With Heart Failure; PREDICT-HF: Prognostic Models Derived in PARADIGM-HF and Validated in ATMOSPHERE and the Swedish Heart Failure Registry to Predict Mortality and Morbidity in Chronic Heart Failure; PROTECT HF: Placebo-Controlled Randomized Study of the Selective A1 Adenosine Receptor Antagonist Rolofylline for Patients Hospitalized With Acute Decompensated Heart Failure and Volume Overload to Assess Treatment Effect on Congestion and Renal Function.

### Treatment of advanced HF in the acute setting

#### Congestion management

Volume overload management remains clinically challenging and may require a combination of several strategies, including higher doses of intravenous loop diuretics, combined diuretic therapy, hypertonic saline, ultrafiltration and peritoneal dialysis.^4^

Although there has been relatively little innovation in this field, recent evidence suggests that remote patient HF monitoring may have potential benefits. Studies of non-invasive home telemonitoring have shown improvements in hospital length of stay and all-cause mortality.^5^Similar results were observed with the implantable CardioMEMS™ HF System, which provides direct pulmonary artery pressure monitoring. CardioMEMS™ proved safe and effective in real-life and post-marketing studies and was also found cost-effective,^6^with reproducible findings across European centers.^7^This promising strategy has translation potential for clinical practice.

#### Cardiogenic shock

Recently, the Society for Cardiovascular Angiography and Intervention (SCAI) has proposed a new consensus statement on the classification of cardiogenic shock (CS) to provide collective language for the different stages and appropriate management of CS. The 5-stage classification allows for a simple hemodynamic definition, providing granularity for the INTERMACS classification.^8^( Figure 2 )

**Figure 2:** – Decision-making algorithm for patients with advanced heart failure. * Interagency Registry for Mechanically Assisted Circulatory Support (INTERMACS) profiles of advanced heart failure. Profile 1: critical cardiogenic shock; Profile 2: progressive decline on inotropic support; Profile 3: stable but IV inotrope dependent; Profile 4: resting symptoms home on oral therapy; Profile 5: exertion intolerant; Profile 6: exertion limited Profile 7: advanced NYHA Class III symptoms. **Cardiogenic shock classification scheme proposed by the Society for Cardiovascular Angiography and Intervention (SCAI). Stage A is “at risk” for cardiogenic shock; stage B is “beginning” shock; stage C is “classic” cardiogenic shock; stage D is “deteriorating”; stage E is “extremis”. Baran, DA, Grines, CL, Bailey, S, et al. SCAI clinical expert consensus statement on the classification of cardiogenic shock. Catheter Cardiovasc Interv. 2019; 94: 29– 37. dooi:10.1002/ccd.28329. IABP: intra-aortic balloon pump; ICD: implantable cardioverter-defibrillator; IV: intravenous; LVAD: left ventricular assist device; MCS: mechanical circulatory support; VA-ECMO: veno-arterial extracorporeal membrane oxygenation; NYHA: New York Heart Association.

In recent years, strategies associated with early intervention in CS, including multidisciplinary team-based management (Shock Team), have highlighted the role of advanced HF specialists in coordinating timely therapeutic decisions.^9^Vasoactive agents are often used to provide hemodynamic and metabolic support, but low-dose combination therapies should be prioritized to avoid further tissue damage. A recent systematic review found no significant difference between vasoactive agents but stressed the importance of early goal-directed therapy, including early hemodynamic stabilization within predefined timelines.^10^Escalating doses of vasoactive agents should prompt consideration of MCS candidacy to prevent irreversible hemodynamic/metabolic derangements of the CS spiral.

Short-term MCS devices are designed to provide uni- or biventricular support for a wide range of conditions, including CS, acute HF, high-risk percutaneous coronary intervention, and cardiac arrest.^11^The most commonly used percutaneous assist systems include intra-aortic balloon pumps (IABP), Impella®, TandemHeart® and veno-arterial extracorporeal membrane oxygenation (VA-ECMO).^4^Despite the preemptive improvement in hemodynamics with these devices, randomized trials have not demonstrated significant reduction in CS mortality.^12^Moreover, recent observational studies hinted at higher rates of adverse events and costs with Impella than IABP.^13^Despite certain limitations, the IABP remains the most widely used MCS device in CS.

In clinical research, the NuPulseCV intravascular ventricular assist system (iVAS) is a novel minimally invasive device that provides long-term ambulatory counterpulsation via a durable pump placed through the subclavian artery and controlled by an external drive unit.^14^The iVAS overcomes many limitations of the IABP and may be a promising option for patients with advanced HF.

## Advanced therapies for HF

The characteristics of candidates for advanced HF therapies, such as HT and left ventricular assist device (LVAD), have changed dramatically over the years, leading to a more complex selection process. Below, we highlight some advances and challenges in the field.

Regarding HT, the treatment of choice for patients with advanced HF,^15^strategies to increase the donor organ pool have been suggested; in fact, in the United States, the United Network for Organ Sharing (UNOS) recently changed its donor organ allocation policy.^16^Given that post-transplant survival is worse with pre-operative VA-ECMO than LVAD, the new system assigns high priority to patients supported with short-tem MCS devices, while stable patients supported with LVAD or inotropes alone are assigned a lower status. In Brazil, some states are making similar changes. Another recent suggestion is the use of predicted heart mass (PHM), rather than body weight as an ideal metric for donor-recipient size matching. Studies have shown that PHM mismatch is a better predictor of primary graft dysfunction and 1-year mortality after HT than weight, height, or body mass index mismatch,^17^and it also predicts right ventricular-pulmonary arterial coupling after HT.^18^Finally, the advent of direct-acting antiviral agents (e.g. sofosbuvir) for treating hepatitis C virus infection has enabled allocation of organs from hepatitis C virus-infected donors to uninfected recipients.^19^

In the field of LVAD, the HeartMate 3™ has been associated with meaningful clinical benefit, with a significant reduction in the rates of ventricular arrhythmias, readmissions, and hemocompatibility-related adverse events (bleeding, thrombosis and stroke).^20^Further technological advances are needed, such as the miniaturization of devices and the development of a truly internalized power system.

Finally, palliative care has proven indispensable in advanced HF management, playing a central role in cases that are not considered eligible for HT or LVAD. Intermittent use of ultrafiltration, peritoneal dialysis, or inotropic infusions can be considered in the hospital, the hospice, or even at home to control symptoms.^1^

## List of participants of the Heart Failure Summit Brazil 2020 / Heart Failure Department - Brazilian Society of Cardiology

Aguinaldo Freitas Junior, Andréia Biolo, Antonio Carlos Pereira Barretto, Antônio Lagoeiro Jorge, Bruno Biselli, Carlos Eduardo Montenegro, Denilson Campos de Albuquerque, Dirceu Rodrigues de Almeida, Edimar Alcides Bocchi, Edval Gomes dos Santos Júnior, Estêvão Lanna Figueiredo, Evandro Tinoco Mesquita, Fabiana G. Marcondes-Braga, Fábio Fernandes, Fabio Serra Silveira, Felix José Alvarez Ramires, Fernando Atik, Fernando Bacal, Flávio de Souza Brito, Germano Emilio Conceição Souza, Gustavo Calado de Aguiar Ribeiro, Humberto Villacorta Jr., Jefferson Luis Vieira, João David de Souza Neto, João Manoel Rossi Neto, José Albuquerque de Figueiredo Neto, Lídia Ana Zytynski Moura, Livia Adams Goldraich, Luís Beck-da- Silva, Luís Eduardo Paim Rohde, Luiz Claudio Danzmann, Manoel Fernandes Canesin, Marcelo Bittencourt, Marcelo Westerlund Montera, Marcely Gimenes Bonatto, Marcus Vinicius Simões, Maria da Consolação Vieira Moreira, Miguel Morita Fernandes da Silva, Monica Samuel Avila, Mucio Tavares de Oliveira Junior, Nadine Clausell, Odilson Marcos Silvestre, Otavio Rizzi Coelho Filho, Pedro Vellosa Schwartzmann, Reinaldo Bulgarelli Bestetti, Ricardo Mourilhe Rocha, Sabrina Bernadez Pereira, Salvador Rassi, Sandrigo Mangini, Silvia Marinho Martins, Silvia Moreira Ayub Ferreira, Victor Sarli Issa.
